# A systematic review of the print media representation of ketamine treatments for psychiatric disorders

**DOI:** 10.1192/bjo.2023.75

**Published:** 2023-06-07

**Authors:** Nicollette L. R. Thornton, Jason Kawalsky, Alyssa Milton, Christiane Klinner, Aaron Schokman, Elizabeth Stratton, Colleen K. Loo, Nick Glozier

**Affiliations:** Central Clinical School, Faculty of Medicine and Health, The University of Sydney, Australia; and Australian Research Council Centre of Excellence for Children and Families over the Life Course, Australia; Central Clinical School, Faculty of Medicine and Health, The University of Sydney, Australia; School of Psychiatry, University of New South Wales, Australia; and Black Dog Institute, Australia; Central Clinical School, Faculty of Medicine and Health, The University of Sydney, Australia; Australian Research Council Centre of Excellence for Children and Families over the Life Course, Australia; and Professor Marie Bashir Centre, Royal Prince Alfred Hospital, Australia

**Keywords:** Antidepressants, ketamine, depressive disorders, media influence, news media

## Abstract

**Background:**

Public and patient expectations of treatment influence health behaviours and decision-making.

**Aims:**

We aimed to understand how the media has portrayed the therapeutic use of ketamine in psychiatry.

**Method:**

We systematically searched electronic databases for print and online news articles about ketamine for psychiatric disorders. The top ten UK, USA, Canadian and Australian newspapers by circulation and any trade and consumer magazines indexed in the databases were searched from 2015 to 2020. Article content was quantitatively coded with a framework encompassing treatment indication, descriptions of prior use, references to research, benefits and harms, treatment access and process, patient and professional testimony, tone and factual basis.

**Results:**

We found 119 articles, peaking in March 2019 when the United States Food and Drug Administration approved esketamine. Ketamine treatment was portrayed in an extremely positive light (*n* = 82, 68.9%), with significant contributions of positive testimony from key opinion leaders (e.g. clinicians). Positive research results and ketamine's rapid antidepressant effect (*n* = 87, 73.1%) were frequently emphasised, with little reference to longer-term safety and efficacy. Side-effects were frequently reported (*n* = 96, 80.7%), predominantly ketamine's acute psychotomimetic effects and the potential for addiction and misuse, and rarely cardiovascular and bladder effects. Not infrequently, key opinion leaders were quoted as being overly optimistic compared with the existing evidence base.

**Conclusions:**

Information pertinent to patient help-seeking and treatment expectations is being communicated through the media and supported by key opinion leaders, although some quotes go well beyond the evidence base. Clinicians should be aware of this and may need to address their patients’ beliefs directly.

## Ketamine in psychiatry

Ketamine has emerged as an efficacious intervention for treatment-resistant depression, and increasingly as a treatment for post-traumatic stress disorder (PTSD).^1^ Many jurisdictions, including the United States Food and Drug Administration (FDA) and the European Medicines Agency, have approved an intranasal spray containing esketamine with established efficacy and safety.^2^ Clinics are also offering off-label racaemic ketamine treatments, and the number of clinics is growing rapidly.^3^ Racaemic ketamine has been used in clinical practice as an anaesthetic and sedative since the 1970s and is listed as an essential medicine by the World Health Organization.^4^ In psychiatry, racaemic ketamine is administered by a variety of routes, including intravenously and via subcutaneous injection. In comparison, esketamine nasal spray is a relatively recent development, and its approval was the first of an antidepressant of a novel mechanism in nearly 30 years.^5^ Unlike racaemic ketamine, esketamine nasal spray for treatment-resistant depression must be co-initiated with a new oral antidepressant.^6^ There is a paucity of information available comparing the efficacy and safety of the two forms of ketamine treatment.

## The role of the media

The media is an important tool in communicating health information to the public, as demonstrated by its influences on health behaviours,^7,8^ including cancer screening rates,^9–12^ treatment choices,^13^ health-related purchasing behaviour^14^ and on vaccination rates.^15^ The media also play a major role in shaping public opinion and health policy;^16,17^ however, health information provided in the news media does not always accurately reflect the current evidence base.^18^ News stories often overemphasise benefits while minimising risks.^19,20^ Given the rapid proliferation of ketamine treatment options and the increasing emphasis on shared decision-making in healthcare, understanding what information patients, carers and the public have about ketamine's benefits and potential harms will help clinicians to address misinformation and manage expectations. To understand this, we undertook a systematic review and quantitative content analysis of print and online media coverage of the use of ketamine in treating psychiatric disorders in four English-speaking jurisdictions up to the time shortly after the first regulatory approvals of esketamine in 2019–2020.

## Method

The protocol was preregistered with Open Science Framework (registration identifier 10.17605/OSF.IO/QT4HJ^21^). We used the electronic databases Factiva and ProQuest Central to capture articles published in print or online by the top ten English-language newspapers, and in consumer and trade periodicals in each of the USA, UK, Canada and Australia. These four countries were selected because they are large, English-speaking countries with relatively available media, and in the case of the UK and USA, their large and influential regulatory bodies, who were early approvers of esketamine nasal spray. The top ten newspapers in each jurisdiction were determined from the most recent publicly available circulation statistics for each country over the search period.^22–25^ Please refer to the Supplementary Material available at https://doi.org/10.1192/bjo.2023.75 for a full list of the 40 newspapers included in the review. All trade or consumer periodical titles (for example, *Pharmaceutical Business Review* or *Rolling Stone Magazine*) that were indexed in either of the two databases used were eligible for inclusion, provided they were published in one of the four countries.

### Eligibility criteria

Articles published from 1 January 2015 to 31 December 2020 that discussed ketamine's therapeutic use for psychiatric disorders were eligible. Articles were ineligible if the primary indication discussed was a neurodegenerative or substance use disorder. Articles published in an academic periodical (as indicated by the title's listing on Ulrich's Web^26^), or those that were under 100 words long, book/television/film/theatre reviews, corrections to previous articles, letters to the editor, classifieds, obituaries or where the primary purpose was to provide financial/investment information, were excluded.

### Search strategy

We used the following search terms: (ketamine OR esketamine OR ‘special K’ OR Spravato OR arketamine OR ‘s-ketamine’ OR ‘r-ketamine’) AND (depress* OR antidepressant OR anxi* OR Trauma OR PTSD OR ‘Post-traumatic stress disorder’ OR bipolar* OR suicid* OR Mood OR ‘Mental Depression’ OR ‘mental illness’).

Searches of both databases were limited to English language results and for the newspaper aspect of the search, the 40 newspapers of interest were specified. This included any variants and websites, e.g. ‘The Daily Mirror’, ‘Daily Mirror’ and ‘mirror.co.uk’. Because of differences in database functionality, other search limits differed between the two databases. In ProQuest Central, source types were limited to ‘magazines’, ‘trade journals’, ‘newspapers’, ‘blogs, podcasts & websites’ and ‘other sources’. Functional limitations necessitated the separation of the newspaper and magazine/trade journal searches in Factiva. For the search of magazine and trade journal articles, the search was limited to the ‘magazines and journals’ source type. Database searches were conducted on 11 February 2021.

Further, for robustness a supplementary search of the websites for each newspaper was conducted with Google Advanced Search. Truncated terms were expanded, syntax modified and the search split up into two separate search phrases to meet the 32 search term limit. The web domain was specified for each of the newspapers, and the process repeated for each one. For example, the two Google Advanced searches of the *New York Post* website were as follows:

1. *ketamine|esketamine|'special K’|Spravato|arketamine|'s-ketamine’|’r-ketamine’ AND depression|depressive|depressed|antidepressant|anxiety|anxious|‘anxiety disorder’|Trauma|PTSD|’Post-traumatic stress disorder’|bipolar|‘bipolar disorder’|‘bipolar depression’ AND site*: https://nypost.com

*AND*

1. *ketamine|esketamine|'special K’|Spravato|arketamine|'s-ketamine’|’r-ketamine’ AND suicide|suicidal|suicidality|Mood|’Mental Depression’|’mental illness’ AND site*: https://nypost.com

Two of the Canadian newspapers, *24 Hours Toronto* and *Metro Toronto*, were discontinued before the search was conducted and therefore did not have active web domains that could be used to complete the Google Advanced supplementary searches. Supplementary searches were completed on 24 February 2021.

### Data processing

Search results from all sources were consolidated into a single spreadsheet and manually inspected for articles that did not meet inclusion criteria based on the article metadata (e.g. place, or date of publication). Duplicate articles published in the same or syndicated to different newspapers were also identified based on these metadata (e.g. the same author and title published on a similar date in two different newspapers) and excluded. Two independent reviewers then conducted a title and abstract screen (N.L.R.T. with C.K. or E.S.). At this point it became apparent that the trade journal articles were not aimed at the lay public and, although included in the registered review protocol, they did not meet the aims of the study and all were excluded from the sample. Articles were eligible for full-text review if either of the two reviewers decided that the article was eligible. A full-text review was conducted by two reviewers (N.L.R.T. and C.K.), and a third reviewer (A.S.) made the final determination regarding any disagreements.

Further duplicates were identified at full-text review and were excluded. This was done by selecting three random sections per article and making comparisons within the context of all other articles remaining in the sample, replicating the method used in plagiarism software. In this final stage, articles were considered to be duplicates if minor changes to wording and grammar were made that did not affect the meaning of the sentences, but the majority of the article remained identical. If the article was duplicated but a small amount of additional text was included (such as a by-line), the longer version of the article was retained.

### Coding and thematic analysis

A quantitative structured coding framework was developed based on the researchers’ knowledge of the treatment and on approaches used in prior research.^27–32^ It encompassed treatment indication, descriptions of prior use, references to research, potential benefits and harms, descriptions of access to treatment and the treatment process, patient and professional testimony, and tone. Free-text items were included to capture positive or negative keywords, phrases that stood out to the coders and/or any suspected factual inaccuracies. The framework was piloted on a sample of articles (*n* = 25) and refined based on discussions with an interdisciplinary team of researchers, including those with lived experience, and medical and allied health professionals. A coding guide was generated and a REDCap^33^ database was built based on the finalised framework to collect and manage study data. One researcher (N.L.R.T.) coded the overall tone of all the articles as ‘mostly positive’, ‘neutral’ (neither positive or negative), ‘mixed’ (approximately equal parts positive and negative) or ‘mostly negative’. Examples of positive tone were support of the use of ketamine in psychiatry, or the portrayal of its use in psychiatry as scientific or medical progress. Examples of a negative tone were opposition to ketamine's therapeutic use or accounts of premature or irresponsible use. Suspected factual inaccuracies were conservatively identified by coders, and subsequently confirmed by senior clinicians with experience using ketamine in both research and clinical settings. A random sample of 25% of articles stratified by pre-rated tone and publication type was then coded against the finalised framework by two reviewers (N.L.R.T. and J.K.). Interrater reliability for main themes was calculated using Cohen's kappa, with *κ* = 0.76 indicating substantial agreement. Once interrater reliability was established, one researcher (N.L.R.T.) coded the remaining articles.

### Analysis

We investigated associations of article tone with expert and patient testimony, reported side-effects, descriptions of prior use, type of ketamine and references to the limitations of evidence regarding long-term safety and efficacy. These associations were evaluated with Fisher's exact test in IBM SPSS Statistics for Windows, version 28.0.^34^

## Results

**Fig. 1:**
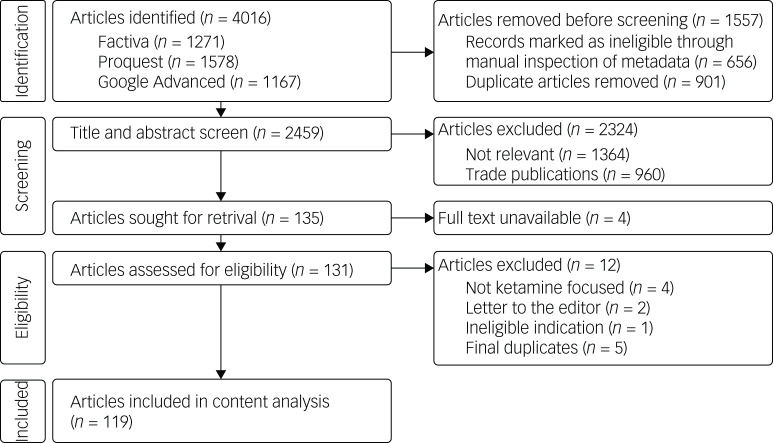
Preferred Reporting Items for Systematic Reviews and Meta-Analyses (PRISMA) diagram demonstrating the article selection process.

The search generated a total of 4016 articles. Manual inspection of metadata found 901 duplicates and 656 articles that did not meet the search criteria. A total of 960 trade publication articles were then removed from the sample, followed by 1364 articles deemed ineligible based on the title and abstract screen. The full text of 131 of the remaining 135 articles was retrieved. Seven of these were excluded following full-text review; four articles did not focus on ketamine, two were ‘letters to the editor’ and one only discussed an ineligible indication. Comparisons between the remaining articles located five additional duplicates, resulting in a final sample of 99 unique newspaper and 20 unique magazine articles (Fig. 1). For a list of all articles included in the final sample, please refer to the Supplementary Material.

**Fig. 2:**
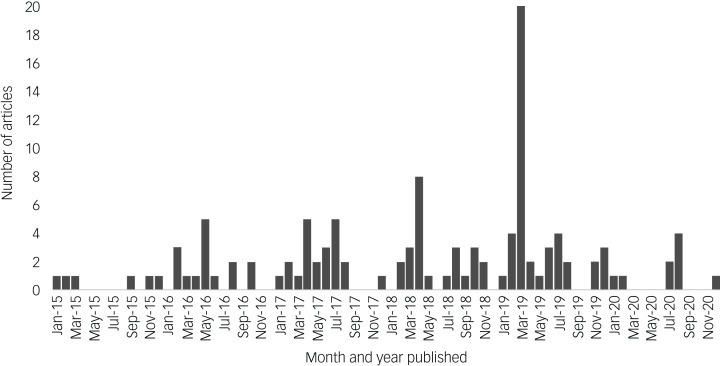
Number of articles published per month from 1 January 2015 to 31 December 2020.

The number of articles published increased year on year, peaking around the FDA approval of esketamine in 2019 before dropping in 2020. Only one article was published between 10 August and 31 December 2020 (Fig. 2).

### Use of ketamine

Ketamine was frequently identified as a drug with a history of illegal use (e.g. recreational or date rape drug; *n* = 101, 84.9%), as well as an anaesthetic (*n* = 91, 76.5%) and/or a veterinary medicine (*n* = 52, 43.7%). Racaemic ketamine was discussed in 64 (53.8%) articles, 50 (42.0%) were about esketamine nasal spray and five (4.2%) were about both ketamine and esketamine.

Almost all articles (*n* = 117, 98.3%) discussed ketamine's use as an antidepressant, 38 (31.9%) discussed its use as an anti-suicidal agent, 13 (10.9%) discussed it as a pharmacotherapy for PTSD and 12 (10.1%) discussed it as a treatment for anxiety and/or obsessive–compulsive disorder. Of the two articles that did not discuss ketamine's use as an antidepressant, one (0.84%) discussed its therapeutic potential in PTSD, and the other its use as an anti-suicidal agent.

### Evidence and safety information

Reference to scientific research was made in 108 (90.8%) articles, and of those, 68 (57.1%) quoted research results. Efficacy was discussed in 51 articles (75% of the 68 articles reporting results; 42.9% of all articles), and this was mostly positive (*n* = 39/51; 76.4%). Efficacy results were reported three times as often as safety results, which were discussed in just 17 (14.3% of total) articles. Approximately 70% (*n* = 12/17) of the safety results quoted were negative.

The benefits of ketamine treatment were reported in 95 (79.8%) articles. The main benefits described were a rapid antidepressant effect (*n* = 87, 73.1%), that it is an effective antidepressant for patients who do not respond to traditional pharmacotherapies (*n* = 36, 30.3%), and explicit claims of ‘superior efficacy’ compared with traditional pharmacotherapies (*n* = 6, 5.0%).

Risks and side-effects were described in 96 (80.7%) articles. Psychotomimetic side-effects (e.g. hallucinations and dissociation) were the most frequently reported (*n* = 78, 65.5%), followed by concerns around addiction or misuse (*n* = 51, 42.9%). Cardiovascular (*n* = 32, 26.9%), urinary (*n* = 27, 22.7%) and cognitive (*n* = 20, 16.8%) side-effects were less frequently reported despite these being some of the primary safety concerns regarding ketamine treatment. No articles discussed medical, psychiatric or pharmaceutical contraindications to ketamine treatment, and only 35 (29.4%) of the articles stated that there were unknowns regarding the long-term safety and/or efficacy of treatments.

### Treatment access and process

The majority of articles (*n* = 78, 65.5%) described ketamine's use in a clinical rather than research setting, with 29 of these (37.1% of articles describing ketamine as currently available in a clinical setting) providing specific information about where the reader could access a ketamine treatment provider, such as the name of the treating clinician and/or practice. Treatment processes most frequently described were the mode of administration (*n* = 67/78, 85.9%), supervised administration of the drug (*n* = 38/78, 48.7%) and frequency of dosing (*n* = 32/78, 41.0%).

### Key opinion leader and patient testimony

Nearly all articles (*n* = 107, 89.9%) included testimony from key opinion leaders (KOLs) such as a medical professional, researcher, regulatory body or pharmaceutical company representative. Of these, 66 (61.7%) articles contained only positive testimony regarding the use of ketamine in psychiatry, 26 articles (24.3%) presented a balanced mix of both positive and negative KOL testimony, nine (8.4%) articles included testimony that was neither in support nor opposition to its use and only six articles (5.6%) presented only negative KOL testimony. Patient testimony and experience with the treatment were included in 32 of the articles (26.9%), of which 29 (90.6%) articles described positive experiences and/or patient testimony. A small minority were mixed or neutral (*n* = 3, 9.4%), and none were negative.

### Overall article tone

Most articles were positive in tone (*n* = 82, 68.9%), 23 articles (19.3%) were an approximately equal mix of positive and negative, 12 (10.1%) were negative and two (1.7%) were neither positive nor negative. Positive article tone was associated with positive expert testimony (Fisher's exact test = 67.784, *P* < 0.001), and articles about generic ketamine were more likely to be positive than articles about esketamine (Fisher's exact test = 5.618, *P* = 0.05). Articles with a positive tone were less likely to acknowledge the limitations of the evidence around the safety and efficacy of long-term ketamine treatments (Fisher's exact test = 7.083, *P* = 0.02). Article tone was not associated with reporting of risks and side-effects or references to ketamine as a recreational or date rape drug.

### Reliability of information

Forty-four (37%) articles contained one or more pieces of inaccurate information. These largely related to treatment efficacy, the longevity of treatment effect, safety information and treatment parameters. Eleven (25%) of these articles included one or more pieces of inaccurate information that were presented as KOL opinion, including direct quotes.

## Discussion

This review indicates that the media portray ketamine treatments for psychiatric disorders in a largely positive light. This is underscored by positive KOL testimony, an emphasis on benefits such as the rapid antidepressant action of the drug and the infrequent reporting of the unknowns regarding long-term safety and efficacy. As expected, the majority of articles reported ketamine's most established psychiatric indications as an antidepressant and as an anti-suicidal agent.^35,36^ The number of articles published spiked around the time that esketamine nasal spray was approved by the FDA in March of 2019, suggesting that this was driven by this process, although more articles discussed racaemic ketamine than esketamine over the period of the search.

**Fig. 3:**
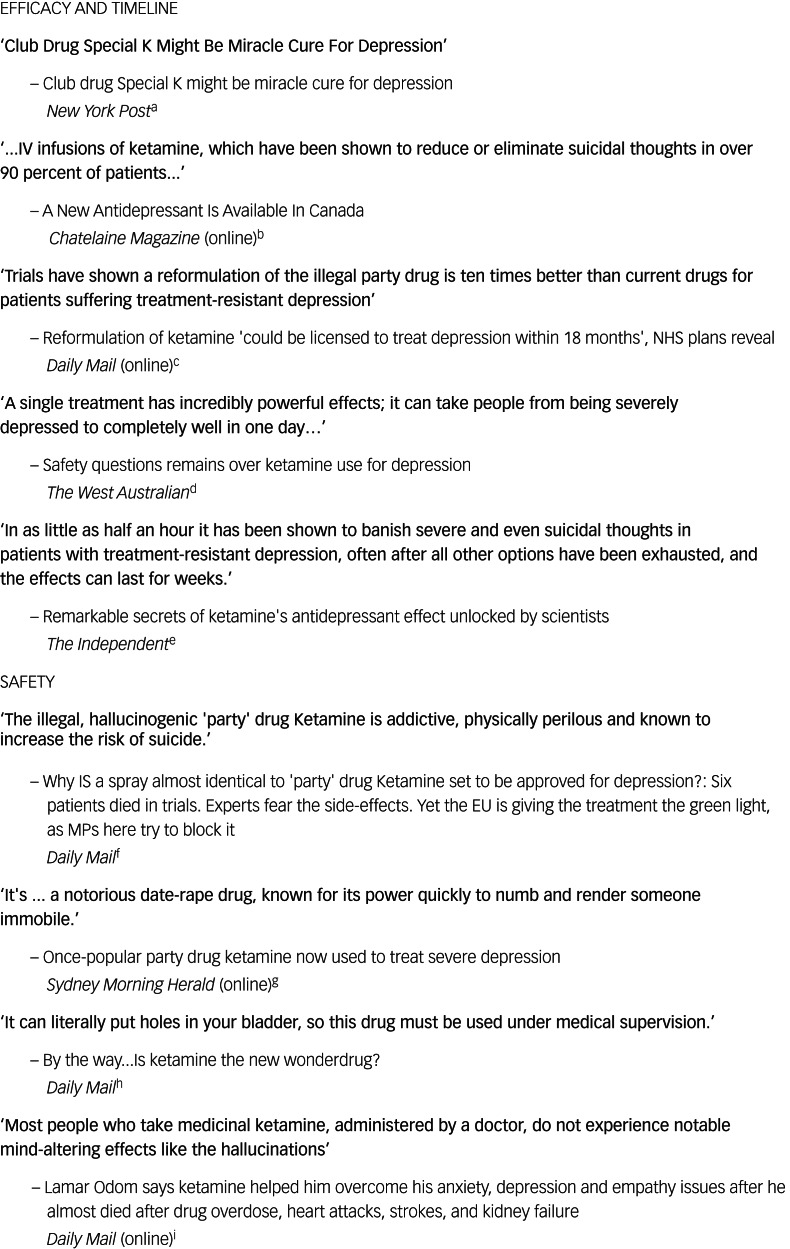
Examples of claims made in articles that are in the journalist's words. ^a37, b38, c39, d40, e41, f42, g43, h44, i45^.

As might be expected, many journalists in major media channels make extravagant claims in their description of both the positive and negative aspects of ketamine (Fig. 3). We do not know the provenance of these; for example, whether from research or company press releases, expert opinion or the journalist's own reading.

Although less frequent than expert testimony, the vast majority of patient's quotes were positive; for example, ‘ … “I immediately felt relief … a lightness of the depression kind of lifting,” says <patient>. “And it all happened in less than an hour.” … ’,^46^ and ‘ … “It's been a lifesaver – literally I feel like it saved my life” … ’.^47^ The experiences of fellow sufferers may be strong determinants of patient's beliefs, as evidenced by word of mouth and online referrals to initial ketamine clinics.

**Fig. 4:**
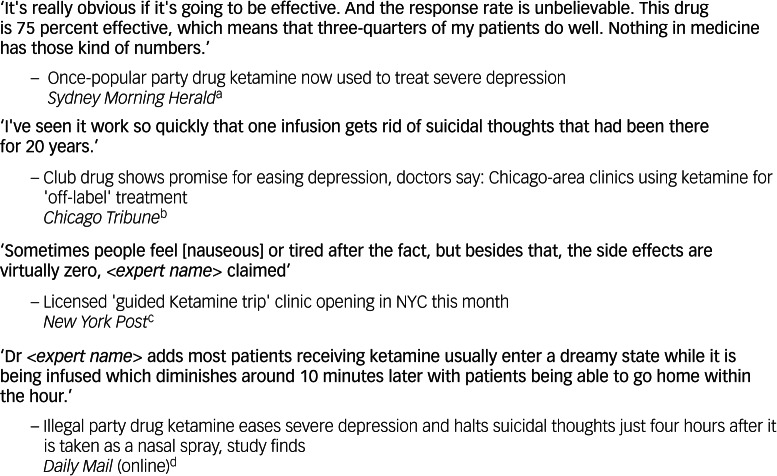
Examples of claims made in articles that are presented as testimony from key opinion leaders and/or medical practitioners. ^a43, b48, c49, d50^.

Echoing prior research investigating online media portrayals of ketamine treatment, KOL testimony was commonly included in articles, and more frequently than patient testimony.^32^ A novel finding is that KOL testimony was strongly associated with the tone of the article and, disconcertingly, some articles included strong statements about treatment efficacy that went well beyond the evidence base (Fig. 4).

These examples of both unattributed and attributed exaggerated claims highlight that clinicians should be cautious when providing expert opinion to journalists. They should consider how their comments may be interpreted by journalists and reported to the public, and the potential impact of that. For example, overly optimistic statements from medical professionals regarding efficacy and/or safety may encourage patients to seek treatments that may not be clinically appropriate. Conversely, exaggeration of the risks may discourage patients from pursuing a treatment that may be suitable for them. A key difficulty is that when providing expert opinion to the media, information may be conveyed over several sentences. As a whole, this may convey a balanced opinion, but selected sentences or phrases may instead be quoted by the journalist. Thus, it is important that each individual statement is accurate on its own. An examination of the total interview given versus what is reported would address the issue of quotation bias, but such data are not typically available. Many professional psychiatric bodies encourage key professional attributes such as advocacy and communication,^51^ but equally have guidelines about what their members should restrict their comments to.^52,53^ At a more extreme end of this spectrum, we have seen medical professionals suspended or deregistered for false medical claims, such as those made about COVID-19 treatments.^54^

A sound understanding of the risks and benefits of a treatment are crucial components of the decision-making process, and therefore it is important that when reporting news about novel medical treatments there is a balanced presentation of the risks and benefits that is backed by the current evidence. The medical profession is increasingly moving away from a purely paternalistic model of care to one where patients are active participants as shared decision makers. To fully participate in shared decision-making, patients need to be adequately informed. Undue stress may be experienced by patients receiving treatment if their experience does not match their expectations. For example, claims regarding ketamine's ‘instant’ effects could cause patients distress if the therapeutic effects are not immediately apparent to them.

In their content analysis of prescription drugs in the UK print news media, Prosser and Clayson^27^ found that newspaper articles often neglected to report the limitations of new medications. Gallagher et al's analysis of online news about ketamine treatments before 2017 found that the rapid antidepressant effects of ketamine were commonly cited, but few reported the lack of evidence of ketamine's effectiveness in the longer term.^32^ Our findings are consistent with this as evidenced by the infrequent reference to the paucity of evidence around the long-term safety and efficacy of ketamine treatments. Prosser and Clayson's study found that benefits are often emphasised whereas risks are underreported.^27^ Our results, however, differ as the side-effects of ketamine treatments were reported in almost all articles, and at an almost identical frequency to the benefits. Nevertheless, there is an asymmetry in the type of side-effects reported, with the psychotomimetic effects and potential for misuse or addiction frequently reported. This contrasts the frequency of reports of side-effects relating to the urinary and cardiovascular systems despite those being some of the core recommended safety monitoring considerations for clinicians treating patients.^55–58^ This difference between reporting on other prescription drugs and novel medical treatments may be explained by ketamine's history of use as a recreational drug.

Our study is distinct from previous similar research. Critically, both previous studies were conducted before esketamine nasal spray was approved to treat treatment-resistant depression. Zhang et al investigated the framing of ketamine in North American print media over the 2000–2015 period.^31^ They did not restrict their search to articles about ketamine's clinical applications in psychiatry, and instead included any articles about ketamine. Their approach investigated the way ketamine as a substance was framed in general terms, and their findings were subsequently discussed in relation to ketamine's clinical use as an antidepressant. Because of the major difference in the scope of the articles included, there are limits to the comparisons that can be made. The authors report that ketamine's potential use as an antidepressant was mentioned in only 12 (27.9%) of the 43 articles in their sample. Only two articles (4.7%) in their entire sample reported ketamine's potential for misuse compared with 42.9% of articles in our study. This is despite ‘abuse’, ‘misuse’, ‘addict’ and ‘overdose’ being four of their seven search terms. Cardiovascular side-effects were reported in 11.4% of their articles, compared with 26.9% in our study. This difference is likely attributable to the earlier study including articles about any topic, rather than being clinical research or health focussed. Gallagher et al^32^ investigated online news media reporting of ketamine depression treatments. They used a 2018 snapshot to identify the 30 top-ranking news websites and searched them for articles published about ketamine's use as an antidepressant from 2000 to 2017. Articles in their study discussed the rapid antidepressant effects of ketamine at a nearly identical frequency (74.2% *v*. 73.1% in our study). KOL were also mentioned often (85% *v*. 89.9% in our study), although they described these as being ‘researchers’ only, whereas our study also included clinicians and pharmaceutical or regulatory body representatives. Patient testimony was not included as frequently (14.4% *v*. 26.9% in our study). This increase may relate to ketamine treatments being more widely available during the period of this study, and therefore more patients treated that were able to provide testimony for articles. Although infrequent in both studies, references to the lack of long-term evidence around ketamine increased in our study (19.6% *v*. 29.4%). The quality and quantity of clinical trial evidence that the FDA based their approval of esketamine on was an issue raised by critics and referred to in some articles in our sample.^42^ The study period of Gallagher et al^32^ did not cover the review and approval of esketamine by any regulatory body. This may explain the difference between the two studies. Side-effects were reported much more frequently in our study (‘just over half’ compared with 80% in our study). It is possible that this is because of additional safety evidence emerging during the period of our study. Potential for misuse was described at a similar frequency compared with the 2015–2017 period (50% *v*. 42.9% in our study).

None of the articles in our study explicitly addressed differences between the use of ketamine in a controlled clinical setting and its use in a recreational context. Future research might investigate the reporting of other emerging novel psychiatric treatments such as MDMA and psilocybin, as these also have a history of recreational use. Investigations of patient experiences of ketamine treatment for psychiatric disorders have highlighted concerns relating to stigma and the public dialogue about ketamine's role in psychiatry.^59^ The news media's emphasis on the illegal use of ketamine may be a contributing factor.

Our study has important limitations and strengths. We did not include other potential sources of health information in our search. Although news media plays a major role in providing information relating to health, new treatments and advances in research to the public, individuals also access this information from other sources. We did not include other news sources such as state broadcasters (e.g. the BBC), social media and online forums, podcasts or television. Audiences have turned away from traditional media sources to alternatives such as social media,^60,61^ which is predominantly the case for younger audiences and those with higher levels of internet literacy and education.^62^ Traditional news outlets continue to adapt their business models to account for these changes.^63^ Much of the online content consumed via social media is generated by traditional media outlets, who often have a presence on social media platforms such as Instagram, Facebook and TikTok.^64,65^ These social media accounts in turn direct traffic to news websites.^66^ That said, a 2022 survey of adults from the USA found that 82% of adults either ‘sometimes’ or ‘often’ access news using digital devices, and news apps and websites were the most favoured method of accessing news digitally.^67^ Bearing all of this in mind, a strength of this study is including both print editions and their online equivalents. Incorporating the online websites of each newspaper into our search strategy allowed us to capture some of the content that might be available to audiences via both non-traditional and digital sources of news.

Our research included articles published by highly circulated newspapers in print as well as their websites. By including highly circulated newspapers in each jurisdiction, we were able to capture the newspaper articles that were likely to have reached the widest audience in each jurisdiction. There are some limitations to this method. It is worth noting that circulation data is counted in different ways by different organisations, sometimes presenting data compiled from multiple sources. Therefore, it may not be representative of the actual circulation of newspaper titles. Some organisations may consider different sub-brands of newspapers to be separate. For example, Sunday editions of newspaper brands might be counted as separate to their ‘daily’ equivalents. This may lead to an overrepresentation of a particular brand in the data-set. We opted to use newspaper circulation data as an objective, proxy measure of which newspaper reached the largest audience in each of the four jurisdictions. This helped us to reduce potential bias that might be introduced by using a purposive sample of newspaper titles based on our knowledge of the markets. Although these data may theoretically be representative of the news that reaches the largest audience, many of the ways that people access and use the news are influenced by algorithms. What information some people may therefore be exposed to may be influenced by their internet search habits and social networks. Investigating the portrayals of ketamine treatments via these non-traditional platforms would certainly be a worthwhile endeavour, but also inherently challenging to conduct in a replicable way. Containing our search to just four relatively large, English-speaking countries is a limitation of the study. These may not reflect the discourse in low-to-middle income countries, or in countries where activities associated with illicit drugs attract more severe penalties (e.g. Singapore or Malaysia). The inclusion of magazine articles is a strength of this study as these articles tend to be longer in format, and therefore potentially provide an opportunity for a more thorough discussion of the topic than newspaper articles. Some of the magazine articles in our final sample are also freely available online without subscription.^38,68^

This is the first systematic search and content analysis of print and online news reports of ketamine treatments for psychiatric disorders undertaken when a form of ketamine treatment has been approved by a medicines regulatory agency to treat a psychiatric disorder. It is apparent from our review that the news media are providing information to the public that might inform patient's treatment decisions. Concerningly, there are instances of inaccurate and exaggerated information being reported purportedly from KOLs with expertise with ketamine, including medical practitioners. Patients may bring very positive views of ketamine such as high expectations of efficacy and the rapidity of its therapeutic effect, and misconceptions should be thoroughly addressed as part of the treatment decision-making process. KOLs should also be mindful of the content of expert comment that they provide to the media, and follow the relevant guidelines for such advocacy.

## Data Availability

The data that support the findings of this study are available from the corresponding author, N.L.R.T., upon reasonable request.

## Supplementary material

For supplementary material accompanying this paper visit http://doi.org/10.1192/bjo.2023.75. click here to view supplementary material
