# Supplementary material for: A systematic review of the print media representation of ketamine treatments for psychiatric disorders
Source: BJPsych Open. 2023 Jun 7;9(4):e104. doi: 10.1192/bjo.2023.75 (PMC10304949; doi:10.1192/bjo.2023.75)
Supplement: Supplementary file 1 [file S2056472423000753sup001.docx]

**A systematic review of the print media representation of ketamine treatments for psychiatric disorders**

NLR Thornton, J Kawalsky, A Milton, C Klinner, A Schokman, E Stratton, CK Loo, N Glozier

**SUPPLEMENTARY MATERIALS**

1. **SEARCHES**
   1. **Newspaper title list**

The forty newspapers were selected based on the most recent, publicly available circulation statistics at the time of the search. These are listed in order of highest circulation to the tenth most circulated newspaper title in the following table:

|  | **United Kingdom ^1^** | **United States ^2^** | **Canada ^3^** | **Australia ^4^** | |
| --- | --- | --- | --- | --- | --- |
| 1 | The Sun | Wall Street Journal | Toronto Star | The Sydney Morning Herald | |
| 2 | The Sun on Sunday | USA Today | The Globe and Mail | The Age | |
| 3 | Daily Mail | New York Times | National Post | The Daily Telegraph | |
| 4 | The Mail on Sunday | Washington Post | 24 Hours Toronto | The Herald Sun | |
| 5 | Daily Mirror | Los Angeles Times | Metro Toronto | The Australian | |
| 6 | Sunday Mirror | New York Post | The Toronto Sun | Australian Financial Review | |
| 7 | Sunday People | Tampa Bay Times | The Vancouver Sun | The Courier Mail | |
| 8 | The Guardian | Chicago Tribune | The Province | Sunday Times | |
| 9 | The Independent | Newsday | The Spectator | The Advertiser | |
| 10 | Daily Express | Star Tribune | Calgary Herald | West Australian | |
|  | *Monthly cross platform data* | *Average weekday print data* | *Weekly cross platform data* | | *Monthly cross platform data* |
|  | *April 2019-March 2020* | *1 January – 31 March 2020* | *2015* | | *1 January to 30 September 2020* |

- 1. **Electronic database search terms**

The following search phrase was used for both the Factiva and Proquest Central databases:

*(ketamine OR esketamine OR "special K" OR Spravato OR arketamine OR "s-ketamine" OR "r-ketamine") AND (depress* OR antidepressant OR anxi* OR Trauma OR PTSD OR "Post-traumatic stress disorder" OR bipolar* OR suicid* OR Mood OR "Mental Depression" OR "mental illness")*

- 1. **Supplementary searches using Google Advanced Search**

Google Advanced Searches were conducted for each newspaper web address using the two search strings below. ‘<URL>’ was replaced by the web address of each newspaper’s website.

1. *ketamine|esketamine|"special K"|Spravato|arketamine|"s-ketamine"|"r-ketamine" AND depression|depressive|depressed|antidepressant|anxiety|anxious|“anxiety disorder”|Trauma|PTSD|"Post-traumatic stress disorder"|bipolar|“bipolar disorder”|“bipolar depression" AND site:<URL>*

*AND*

1. *ketamine|esketamine|"special K"|Spravato|arketamine|"s-ketamine"|"r-ketamine" AND suicide|suicidal|suicidality|Mood|"Mental Depression"|"mental illness" AND site: <URL>*
2. **PICOS TABLE**

Not applicable to this review

1. **LIST OF MEDIA ARTICLES INCLUDED IN THE REVIEW**

| **Article title** | **Publication title** | **Type** | **Date Published** |
| --- | --- | --- | --- |
| Club drug shows promise for easing depression, doctors say: Chicago-area clinics using ketamine for 'off-label' treatment | Chicago Tribune | Newspaper | 04/04/2018 |
| Ray of light in ketamine | Herald Sun | Newspaper | 23/07/2017 |
| FDA approves new nasal spray for untreatable depression | National Post | Newspaper | 06/03/2019 |
| Ketamine: The illicit party psychedelic that promises to heal depression | National Post | Newspaper | 15/05/2019 |
| UK healthcare cost agency rejects J&J's nasal spray for depression | National Post | Newspaper | 28/01/2020 |
| A party drug instantly cured my depression | New York Post | Newspaper | 28/04/2016 |
| This party drug could help treat depression | New York Post | Newspaper | 03/05/2017 |
| Club drug Special K might be miracle cure for depression | New York Post | Newspaper | 31/10/2018 |
| FDA approves ketamine's 'chemical cousin' to treat depression | New York Post | Newspaper | 06/03/2019 |
| This party drug is a breakthrough in depression treatment | New York Post | Newspaper | 18/03/2019 |
| Anti-depressant Esketamine could be approved for use in UK | New York Post | Newspaper | 13/07/2019 |
| Licensed 'guided Ketamine trip' clinic opening in NYC this month | New York Post | Newspaper | 05/08/2020 |
| Can We Stop Suicides? | New York Times | Newspaper | 30/11/2018 |
| F.D.A. Panel Recommends New Depression Treatment | New York Times | Newspaper | 12/02/2019 |
| Fast-Acting Depression Drug, Newly Approved, Could Help Millions | New York Times | Newspaper | 05/03/2019 |
| Doctors Welcome New Depression Drug Cautiously | New York Times | Newspaper | 08/03/2019 |
| Costly Depression Drug Accepted for Veterans, Despite Safety Concern | New York Times | Newspaper | 24/06/2019 |
| Approved nasal spray gives hope to patients with severe depression | Newsday | Newspaper | 06/03/2019 |
| Pros cons of new drug for depression | Star Tribune | Newspaper | 21/04/2019 |
| Medical clinics promote ketamine injections as treatment for depression | Sydney Morning Herald | Newspaper | 17/02/2015 |
| Once-popular party drug ketamine now used to treat severe depression | Sydney Morning Herald | Newspaper | 03/02/2016 |
| Doctor under 'commercial pressure' to prescribe ketamine | Sydney Morning Herald | Newspaper | 24/05/2018 |
| Nasal spray is new anti-depressant option to combat US suicide risks | Sydney Morning Herald | Newspaper | 10/08/2020 |
| Depression researchers stop ketamine nasal spray trial because of psychotic-like effects | The Age | Newspaper | 15/03/2018 |
| Party drug Ketamine offers clinical boon for depression | The Australian | Newspaper | 05/05/2016 |
| Severe depression cure - illness could be treated with Ketamine - but advice comes warning | The Daily Express | Newspaper | 06/04/2017 |
| Horse tranquilliser ketamine can treat depression and suicidal thoughts in humans | The Daily Express | Newspaper | 16/04/2018 |
| Reformulation of ketamine 'could be licensed to treat depression within 18 months', NHS plans reveal | The Daily Express | Newspaper | 19/10/2018 |
| 'I couldn't get depressed if I tried': Sons And Daughters star Rowena Wallace reveals she's taking horse tranquilizer and sometime party drug KETAMINE for her mental health issues | The Daily Mail | Newspaper | 16/03/2015 |
| Could ketamine be prescribed to treat depression? Party drug hailed as the 'next big thing in psychiatry' | The Daily Mail | Newspaper | 03/02/2016 |
| Secret of HOW ketamine eases depression is unlocked by scientists, raising hopes of new, faster-acting antidepressants | The Daily Mail | Newspaper | 05/05/2016 |
| Nasal spray that beats the blues in just minutes | The Daily Mail | Newspaper | 30/10/2016 |
| Could a ketamine vaccine prevent PTSD? Scientists seem to think so | The Daily Mail | Newspaper | 09/02/2017 |
| Illegal party drug ketamine should be used to treat depression as study finds the horse tranquiliser helps those who don't respond to conventional treatments | The Daily Mail | Newspaper | 06/04/2017 |
| Ketamine is a safe and powerful cure for elderly people with depression, study claims: 50% of patients 'cured of symptoms after taking the club drug' | The Daily Mail | Newspaper | 26/07/2017 |
| Could the illegal party drug ketamine ease depression? Horse tranquilizer improves sufferers' sleep and interest | The Daily Mail | Newspaper | 16/08/2017 |
| Why club drug ketamine could treat depression: Horse tranquilizer quells suicidal thoughts in sufferers, new study explains | The Daily Mail | Newspaper | 16/12/2017 |
| Illegal party drug ketamine eases depression in just 30 minutes: Horse tranquiliser relieves the mental health disorder by preventing brain signals 'overfiring', study finds | The Daily Mail | Newspaper | 17/02/2018 |
| Researchers looking to find new depression treatment forced to abort ketamine nasal spray trial after patients suffered psychotic side-effects when taking 'huge hits' of the horse tranquiliser drug | The Daily Mail | Newspaper | 16/03/2018 |
| 'I went from wanting to kill myself to being fine': Illegal party drug ketamine cured a suicidal woman's depression after just FOUR treatments | The Daily Mail | Newspaper | 12/04/2018 |
| Illegal party drug ketamine eases severe depression and halts suicidal thoughts just four hours after it is taken as a nasal spray, study finds | The Daily Mail | Newspaper | 16/04/2018 |
| Scientists test ketamine on CHILDREN with treatment-resistant depression - and say the drug cleared up their symptoms in two weeks | The Daily Mail | Newspaper | 03/08/2018 |
| Ketamine may treat depression, pain and addiction - by triggering the brain's opioid response, study finds | The Daily Mail | Newspaper | 29/08/2018 |
| By the way...Is ketamine the new wonderdrug? | The Daily Mail | Newspaper | 11/09/2018 |
| Party drug ketamine 'to treat depression' | The Daily Mail | Newspaper | 16/10/2018 |
| Ketamine infusions could work better than antidepressants for patients with hard-to-treat depression, anxiety and substance abuse | The Daily Mail | Newspaper | 17/01/2019 |
| Nasal spray made from KETAMINE wins FDA panel's backing in a major step toward using the party drug to treat depression | The Daily Mail | Newspaper | 13/02/2019 |
| Depression ages the brain, first human trials suggest: Damage appears 10 years early in people with the mental health condition - but it could be treated with ketamine | The Daily Mail | Newspaper | 15/02/2019 |
| Nose spray that could help treat depression | The Daily Mail | Newspaper | 16/04/2019 |
| Lamar Odom says ketamine helped him overcome his anxiety, depression and empathy issues after he almost died after drug overdose, heart attacks, strokes, and kidney failure | The Daily Mail | Newspaper | 05/06/2019 |
| A controversial Ketamine-like nasal spray could be approved to treat depression in the UK because doctors say it works within just hours | The Daily Mail | Newspaper | 17/07/2019 |
| Why IS a spray almost identical to 'party' drug Ketamine set to be approved for depression?: Six patients died in trials. Experts fear the side-effects. Yet the EU is giving the treatment the green light, as MPs here try to block it | The Daily Mail | Newspaper | 19/11/2019 |
| Drug derived from horse tranquiliser Ketamine is licensed for use treating severe depression in the UK | The Daily Mail | Newspaper | 21/12/2019 |
| NHS watchdog rejects use of ketamine-like nasal spray as antidepressant despite hype over treatment | The Daily Mail | Newspaper | 08/02/2020 |
| US health chiefs approve antidepressant nasal spray derived from party drug ketamine to treat suicidal people | The Daily Mail | Newspaper | 04/08/2020 |
| Manic depressives could be treated with KETAMINE say medical experts | The Daily Mirror | Newspaper | 05/02/2016 |
| New way to use club drug ketamine 'could help treat depression without any harmful side-effects' | The Daily Mirror | Newspaper | 05/05/2016 |
| 2 MINUTES ON...: Radical treatment for depression | The Daily Mirror | Newspaper | 02/06/2017 |
| The depression drug that produces results in hours | The Daily Mirror | Newspaper | 11/11/2019 |
| Warning over ketamine use for depression | The Daily Telegraph | Newspaper | 15/12/2015 |
| Ketamine offers new hope for patients with severe depression | The Globe and Mail | Newspaper | 16/02/2017 |
| Depressed? Your doctor might soon prescribe ketamine | The Guardian | Newspaper | 03/03/2016 |
| First 'gold-standard' trial of ketamine's anti-depressant effects launched | The Guardian | Newspaper | 16/08/2016 |
| If ketamine helps treat depression, why can't doctors prescribe it? | The Guardian | Newspaper | 03/04/2017 |
| Ketamine could help thousands with severe depression | The Guardian | Newspaper | 02/06/2017 |
| 'I can stop and breathe': the people taking ketamine for depression | The Guardian | Newspaper | 02/06/2017 |
| Ketamine-related drug could be 'watershed' in treating depression | The Guardian | Newspaper | 08/03/2019 |
| Ketamine: can it really be an antidepressant? | The Guardian | Newspaper | 23/03/2019 |
| The controversial ketamine-like drug that Trump is pushing on veterans | The Guardian | Newspaper | 18/06/2019 |
| Ketamine-like drug for depression could get UK licence within the year | The Guardian | Newspaper | 13/07/2019 |
| Ketamine-like drug for depression gets UK licence | The Guardian | Newspaper | 20/12/2019 |
| Ketamine could be used as anti-depressant | The Independent | Newspaper | 15/09/2015 |
| Ketamine to undergo $2m trial as treatment for depression | The Independent | Newspaper | 10/11/2015 |
| Ketamine could hold key to side-effect free antidepressant, scientists reveal | The Independent | Newspaper | 04/05/2016 |
| Ketamine helps patients with severe depression 'when nothing else works', doctors say | The Independent | Newspaper | 05/04/2017 |
| Botox and ketamine could help treat depression, study finds | The Independent | Newspaper | 04/05/2017 |
| Ketamine has 'truly remarkable' effect on depression and is effective in elderly patients, scientists say | The Independent | Newspaper | 25/07/2017 |
| Remarkable secrets of ketamine's antidepressant effect unlocked by scientists | The Independent | Newspaper | 15/02/2018 |
| Psychotic-like effects halt spray trial for depression: CLINICAL TIME | The Independent | Newspaper | 16/03/2018 |
| Is ketamine the new wonder drug for depression? As someone who's struggled with traditional methods, I'm hopeful - but cautiously so | The Independent | Newspaper | 16/04/2018 |
| Ketamine nasal spray rapidly relieves depression and suicidal thoughts, finds trial | The Independent | Newspaper | 16/04/2018 |
| Ketamine antidepressant could bring opioid-like addiction risks, study warns | The Independent | Newspaper | 29/08/2018 |
| Ketamine-based depression drug gets US approval | The Independent | Newspaper | 07/03/2019 |
| GIDDY UP! Party drug and anaesthetic ketamine 'TWICE as effective as standard drugs at treating depression' | The Sun | Newspaper | 06/04/2017 |
| HIGH HOPES Party drug ketamine can 'help beat depression and suicidal thoughts in FOUR HOURS' | The Sun | Newspaper | 16/04/2018 |
| GOOD TRIP? What is Ketamine , what are the side effects and can the drug help battle depression ? | The Sun | Newspaper | 12/07/2018 |
| SPECIAL K Party drug Ketamine may soon be prescribed to treat depression in UK as docs say nasal spray work in just hours | The Sun | Newspaper | 13/07/2019 |
| WONDER DRUG? Ketamine nose spray that 'may be able to RELIEVE suicidal thoughts' approved by the FDA | The Sun | Newspaper | 06/08/2020 |
| Biggest breakthrough since antidepressants' is turning lives around in Ottawa | The Toronto Sun | Newspaper | 25/01/2017 |
| FDA allows treatment of depression with club drug's cousin | Toronto Star | Newspaper | 06/03/2019 |
| The FDA has approved Spravato nasal spray to treat major depressive disorder | USA Today | Newspaper | 06/03/2019 |
| 'It saved my life': Relative of party drug 'Special k' could revolutionize depression treatment | USA Today | Newspaper | 07/03/2019 |
| Health & Wellness: Researchers Study New Ways to Treat Suicide Risk | Wall Street Journal | Newspaper | 21/06/2016 |
| FDA Approves Controversial Drug for Depression; Nasal spray branded Spravato by manufacturer Johnson & Johnson is close chemical relation to ketamine | Wall Street Journal | Newspaper | 06/03/2019 |
| Nasal spray of party drug shows promise as fast-acting antidepressant, researchers say | Washington Post | Newspaper | 20/04/2018 |
| Psychedelic past of FDA-approved antidepressant | Washington Post | Newspaper | 12/03/2019 |
| Depression drug trial | West Australian | Newspaper | 16/08/2016 |
| Safety questions remains over ketamine use for depression | West Australian | Newspaper | 28/07/2017 |
| Ketamine and nangs used to treat depression | West Australian | Newspaper | 08/07/2020 |
| A New Antidepressant Is Available In Canada | Chatelaine | Magazine | 03/12/2020 |
| FDA Approves Johnson & Johnson's Ketamine-Derived Drug For Treatment-Resistant Depression | Forbes | Magazine | 05/03/2019 |
| The ketamine club | Los Angeles Magazine | Magazine | 01/07/2017 |
| Ketamine spray for depression? | New Scientist | Magazine | 12/05/2018 |
| Reason for optimism | New Scientist | Magazine | 16/03/2019 |
| Could Ketamine stop suicide? | New Statesman | Magazine | 23/01/2015 |
| A Quick Hit for Depression | Psychology Today | Magazine | 01/11/2018 |
| The FDA Has Approved a Drug Similar to Ketamine to Treat Depression | Rolling Stone | Magazine | 06/03/2019 |
| Is Esketamine the Game-Changer for Depression We Want? | Rolling Stone | Magazine | 11/03/2019 |
| HOT MEDICINAL HELP ZOOM KETAMINE CLINICS | Rolling Stone | Magazine | 01/07/2020 |
| How Ketamine really fights depression | Science News | Magazine | 28/05/2016 |
| New drug approved for severe depression | Science News | Magazine | 21/12/2019 |
| Donald Trump Thinks He Can Stop Veteran Suicides With Ketamine | Spin | Magazine | 21/08/2019 |
| Trump Orders 'a Lot' of Ketamine for Depressed Veterans | The Atlantic | Magazine | 23/08/2019 |
| Sniffing at a new solution; Depression and its treatment | The Economist | Magazine | 15/10/2016 |
| Ketamine treatment | The Economist | Magazine | 16/03/2019 |
| 'Club drug' variant wins FDA approval | The Weekly Standard | Magazine | 12/03/2019 |
| The club drug Ketamine may treat depression-but the risks could be big | Time | Magazine | 20/03/2017 |
| Hope from a strange source | Time | Magazine | 07/08/2017 |
| Victims Find Relief Through Ketamine | USA Today | Magazine | 01/02/2019 |

1. **ADDITIONAL SENSITIVITY ANALYSES OR ADDITIONAL ANALYSES**

Not applicable to this review

1. **PUBLICATION BIAS**

Not applicable to this review

1. **QUALITY ASSESSMENT**

Not applicable to this review

1. **REFERENCES FOR SUPPLEMENTARY MATERIAL**

1. Publishers Audience Measurement Company Ltd. Individual Brand Reach Tables - Newsbrands PAMCo 3 2020. London, UK: Publishers Audience Measurement Company Ltd; 2020 Apr.

2. Turvill W. Top ten US newspaper circulations: Biggest print titles have lost 30% of sales since 2016 election 2020 Oct 22 [Available from: <https://pressgazette.co.uk/media-audience-and-business-data/media_metrics/top-ten-us-newspaper-circulations-biggest-print-titles-have-lost-30-of-sales-since-2016-election/>.

3. News Media Canada. Circulation Report: Daily Newspapers 2015. 2016. Available from: <https://nmc-mic.ca/wp-content/uploads/2016/06/2015-Daily-Newspaper-Circulation-Report-REPORT_FINAL.pdf>.

4. Article No. 8569: New Roy Morgan Cross-Platform Audience results show continued growth for Australia’s leading mastheads [press release]. Melbourne, AU: Roy Morgan2020 18 Nov.
